# Overcoming Multidrug Resistance in Human Cancer Cells by Natural Compounds

**DOI:** 10.3390/toxins2061207

**Published:** 2010-05-28

**Authors:** Tomohiro Nabekura

**Affiliations:** Faculty of Pharmaceutical Sciences, Niigata University of Pharmacy and Applied Life Sciences, Higashi-jima, Akiha-ku, Niigata 956-8603, Japan; Email: nabe@nupals.ac.jp; Tel.: +81-250-25-5000; Fax: +81-250-25-5021

**Keywords:** P-glycoprotein, ABC transporter, anticancer drug, multidrug resistance, phytochemical, cancer chemoprevention

## Abstract

Multidrug resistance is a phenomenon whereby tumors become resistant to structurally unrelated anticancer drugs. P-glycoprotein belongs to the large ATP-binding cassette (ABC) transporter superfamily of membrane transport proteins. P-glycoprotein mediates resistance to various classes of anticancer drugs including vinblastine, daunorubicin, and paclitaxel, by actively extruding the drugs from the cells. The quest for inhibitors of anticancer drug efflux transporters has uncovered natural compounds, including (-)-epigallocatechin gallate, curcumin, capsaicin, and guggulsterone, as promising candidates. In this review, studies on the effects of natural compounds on P-glycoprotein and anticancer drug efflux transporters are summarized.

## 1. Introduction

Cancer is the leading cause of death in Japan, and the second cause of death in the United States. Carcinogenesis is generally recognized as a multistep process in which a normal cell acquires specific phenotypic characteristics including self-sufficiency in growth signals, evasion of apoptosis, limitless replicative potential, sustained angiogenesis and the ability to undergo tissue invasion and metastasis [1].

Cancer chemotherapy is the treatment of cancer using anticancer drugs with the aim to achieve and maintain remission. Multidrug resistance is a phenomenon of resistance of tumors to chemically unrelated anticancer drugs, and is one of the most formidable challenges in the field of cancer chemotherapy. Multidrug resistance can have many causes, but the most commonly encountered mechanism in the laboratory is the increased efflux of cytotoxic drugs by energy-dependent transporters. P-glycoprotein belongs to the large ATP-binding cassette (ABC) transporter superfamily of membrane transport proteins, and was the first member to be identified [2,3,4]. P-glycoprotein mediates resistance to a variety of pharmacologically unrelated anticancer drugs, such as vinblastine, vincristine, daunorubicin, epirubicin, etoposide, imatinib, irinotecan, and paclitaxel [2,3,4].

Cancer prevention is aimed to reduce the cancer mortality by reduction in the incidence of the carcinogenic process [5,6,7]. Fruits and vegetables are excellent sources of fiber, vitamins, and minerals, but they also contain various phytochemicals that may provide substantial health benefits beyond basic nutrition [5,6,7]. Phytochemicals are non-nutritive components in the plant-based diet that possess substantial anticarcinogenic properties. (-)-Epigallocatechin gallate, curcumin, and capsaicin serve as a wide variety of naturally occurring phytochemicals with cancer chemopreventive activity [5,6,7].

Recently, several studies have documented the ability of chemopreventive phytochemicals to increase the sensitivity of cancer cells to anticancer drugs. Natural compounds including flavonoids such as quercetin, morin, and (-)-epigallocatechin gallate, curcumin, capsaicin, and [6]-gingerol can reverse the multidrug resistance by the inhibition of anticancer drug efflux transporter P-glycoprotein and other ABC transporters [8,9,10,11]. In this review, studies on the effects of natural compounds on ABC transporters are summarized, for the future development of safe and effective inhibitors of ABC transporters to overcome multidrug resistance in human cancer.

## 2. Multidrug Resistance and Cancer Chemoprevention

### 2.1. P-glycoprotein

P-glycoprotein is encoded by *MDR1*, also referred to as *ABCB1* [2,3,4]. The human *MDR1* (*ABCB1*) gene is located on chromosome 7q21. It consists of 28 exons, which encode a 1280-amino acid glycoprotein. The molecular structure of P-glycoprotein consists of two bundles of six transmembrane helices that form a drug-binding cavity with two ATP-binding sites [2,3,4].

Besides anticancer agents, various clinically important drugs, including digoxin, verapamil, cyclosporin A, tacrolimus, quinidine, talinolol, erythromycin, ivermectin, fexofenadine, progesterone and saquinavir, are substrates of P-glycoprotein [2,3,4]. P-glycoprotein is also expressed in normal tissues. It is abundant in the apical membranes of many pharmacologically important epithelial barriers, such as the intestinal epithelium, renal proximal tubular epithelium and the blood-brain barrier [2,3,4]. Therefore, it is considered that P-glycoprotein plays very important roles in the absorption, distribution and elimination of many commonly used drugs, and thus determines the efficacy and toxicity of drugs. For instance, P-glycoprotein at the apical membrane of enterocytes acts as a biochemical barrier and restricts the absorption of orally administered drugs.

Recently, pharmacotherapy has become more diversified and complex, and clinical practice frequently involves the concomitant use of multiple medications. As a consequence, drug-drug interactions are more likely to occur. The modulation of P-glycoprotein activity by a P-glycoprotein inhibitor can be an important factor in modifying the bioavailability and tissue distribution of P-glycoprotein substrates. Many clinically significant P-glycoprotein-mediated drug-drug interactions have been reported [12].

In addition to drug-drug interactions, food-drug interactions can also occur. It is now established that foods can have pronounced impacts on drug absorption, disposition and elimination [13,14]. Grapefruit juice and the mild antidepressant St. John's wort (*Hypericum perforatum*) is a well known example of food-drug interactions [13,14].

### 2.2. Chemopreventive phytochemicals

Epidemiological and experimental studies have highlighted the ability of micronutrients in fruits and vegetables to reduce the risk of cancer [5,6,7]. Recently, attention has focused on phytochemicals, non-nutritive components of a plant-based diet that possess cancer preventive properties [5,6,7].

The signaling pathways that govern cell proliferation, survival and oncogenesis are of prime interest in the biology of cancer. The nuclear factor κB (NF-κB) transcription factors (p50/p105 (NF-κB1), p52/p100 (NF-κB2), RelA (p65), RelB, c-Rel) and the pathways that control NF-κB activation are best known for their role in inflammatory responses, but also critical to many physiological and pathophysiological responses, including cell differentiation, adhesion, survival, and apoptosis [15,16]. In most resting cells, NF-κB is sequestered in the cytoplasm by binding to the inhibitory IκB proteins. NF-κB is activated by a variety of stimuli such as carcinogens, inflammatory agents including phorbol esters, bacterial endotoxins, and tumor necrosis factor (TNF). These stimuli promote dissociation of IκB through phosphorylation, ubiquitination and proteasome-mediated degradation. This process unmasks the nuclear localization sequences of NF-κB, which then accumulates in the nucleus, binds to κB regulatory elements and activates target genes [15,16]. Chemopreventive phytochemicals, such as curcumin and capsaicin, are known to block the NF-κB activation process [5,6,7].

Wnts are an evolutionarily conserved family of growth factors that are essential for a wide array of developmental and physiological processes [17,18]. The activity of the Wnt/β-catenin signaling pathway is dependent upon the amount of β-catenin in the cytoplasm. In the absence of Wnt ligands, the cytoplasmic β-catenin level is kept low through continuous ubiquitin-proteasome-mediated degradation, by a multiprotein destruction complex containing axin, adenomatous polyposis coli (APC), and glycogen synthase kinase 3 (GSK3). Upon binding of Wnts to the receptor complex Frizzled (Fz) and LDL receptor-related proteins (LRP), a signal is transduced to activate the cytoplasmic phosphoprotein Dishevelled (Dvl). Activated Dvl inhibits GSK3, thus preventing phosphorylation of β-catenin. Consequently, β-catenin escapes proteasomal degradation, accumulates in the cytoplasm and subsequently translocates to the nucleus. In the nucleus, β-catenin activates target genes in cooperation with transcription factors T-cell factor (TCF) and lymphoids enhancer factor (LEF) [17,18]. Cyclin D1 and c-Myc, cell proliferation and apoptosis regulators, are well known targets of Wnt/β-catenin signaling [17,18]. Mutation of the APC gene occurs in the majority of sporadic colorectal cancers, as well as familial adenomatous polyposis [17,18]. Chemopreventive phytochemicals, such as curcumin and (-)-epigallocatechin gallate, are known to block the Wnt/β-catenin signaling pathway [19,20,21,22]. Therefore, NF-κB and Wnt signaling pathways are important molecular targets of cancer prevention by natural compounds.

### 2.3. Overcoming multidrug resistance

**Figure 1:**
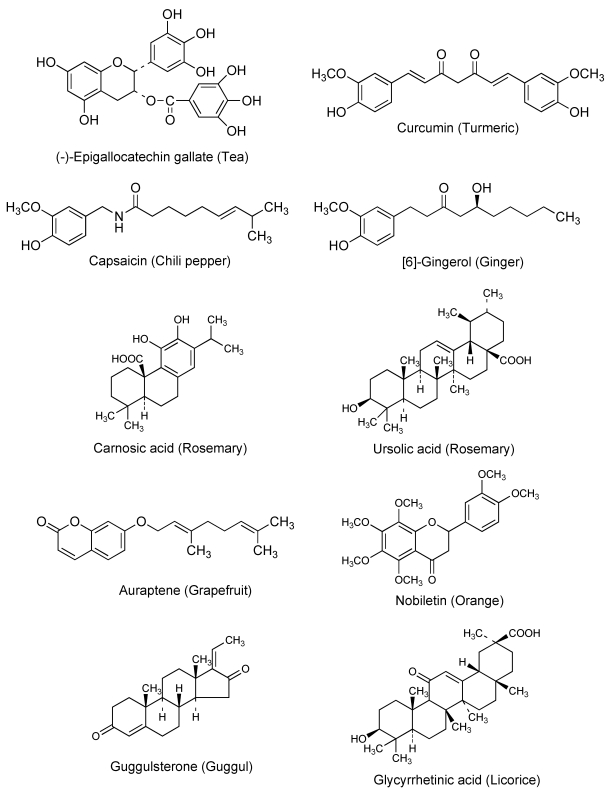
Chemical structures and source of chemopreventive phytochemicals.

Cancer chemotherapy is usually a marginal proposition in the sense that the maximum dose tolerated by the patient is often barely sufficient to kill a useful percentage of the cancer cells. Relatively small increases in drug resistance in cancer cells are thus sufficient to render the drug ineffective. ABC transporters are expressed at cancer cell membranes and can cause multidrug resistance [2,3,4]. Therefore, ABC transporters seem to be good targets for circumventing multidrug resistance. Since the discovery of P-glycoprotein in the 1970s, various attempts and clinical trials have been carried out using a P-glycoprotein inhibitor such as verapamil or cyclosporine to overcome multidrug resistance. However, due to the side effects or ineffectiveness of these compounds, a successful outcome has not been achieved [2,3,4]. In general, natural dietary phytochemicals from foods, herbs, and dietary supplements are thought to be less toxic to the body than medical drugs. The quest for inhibitors of anticancer drug exporters has uncovered natural compounds including quercetin, (-)-epigallocatechin gallate, curcumin, capsaicin, and [6]-gingerol, as promising candidates [8,9,10,11]. Figure 1 shows the chemical structures and source of dietary chemopreventive phytochemicals that can inhibit P-glycoprotein. Cancer chemopreventive and chemosensitizing properties of several natural products are described in greater detail in the following sections.

## 3. P-glycoprotein and Chemopreventive Phytochemicals

### 3.1. Tea

Tea (*Camellia sinensis*) consumption has been shown to reduce the risk of tumor formation at different organ sites including the skin, oral cavity, esophagus, stomach, intestine, lung, liver, pancreas, mammary gland, urinary bladder, and prostate [5,6,7]. (-)-Epigallocatechin gallate (EGCG), a major water-extractable constituent of tea, has been presumed to be the active compound for the cancer preventive effects. A cup of green tea (2.5 g of dried green tea leaves brewed in 200 mL of water) may contain 90 mg of EGCG, and similar or a slightly smaller amount of (-)-epigallocatechin, about 20 mg each of (-)-epicatechin gallate and (-)-epicatechin [23].

Nomura *et al*. [24] reported the inhibitory activity of EGCG on NF-κB pathway in mouse epidermal cells. In the JB6 mouse epidermal cells, a tumor promoter 12-*O*-tetradecanoylphorbol-13-acetate (TPA) markedly induced NF-κB activation. EGCG blocked TPA-induced phosphorylation of IκB resulted in the inhibition of NF-κB activity [24]. It was also reported that EGCG inhibits ultraviolet (UV)-induced phosphorylation of IκB and activation of NF-κB in human epidermal keratinocytes [25].

Dashwood and colleagues have reported the inhibition of heterocyclic amine 2-amino-1-methyl-6-phenylimidazo[4,5-*b*]pyridine (PhIP)-induced formation of intestinal polyps in Apc^min^ mouse by tea [26]. They subsequently revealed the inhibition of Wnt/β-catenin signaling by tea constituents using β-catenin/TCF reporter construct [21]. β-Catenin/TCF reporter activity in human embryonic kidney 293 (HEK293) cells was inhibited by 25 μM EGCG [20]. Kim *et al*. [22] reported the inhibition of Wnt/β-catenin signaling by EGCG in human invasive breast cancer cells. EGCG induced the HBP1 transcriptional repressor, a suppressor of Wnt signaling, thus reducing both tumorigenic proliferation and invasiveness of breast cancer cells [22].

In addition to the anti-carcinogenic effects of EGCG, we have revealed the inhibitory effects of EGCG and other tea catechins on the function of P-glycoprotein using human carcinoma KB-C2 cells and fluorescent P-glycoprotein substrates daunorubicin and rhodamine 123 [10]. KB-C2 is a multidrug-resistant human epidermal carcinoma cell line that over-expresses P-glycoprotein [27,28]. Daunorubicin and rhodamine 123 have often been used in studies of P-glycoprotein-mediated transport. Daunorubicin is also reported to be a substrate of multidrug resistance protein 1 (MRP1). However, MRP1 is scarcely found in KB-C2 cells, and its involvement in daunorubicin accumulation in KB-C2 cells is negligible. We showed that tea catechins (10–100 μM) increased the cellular accumulation of daunorubicin and rhodamine 123 in the order of (-)-epigallocatechin < (-)-epicatechin gallate < EGCG [10]. (-)-Epicatechin had no effects up to 300 μM. We also measured the partition coefficients P_oct_ of tea catechins between *n*-octanol and phosphate-buffered saline (PBS). The P_oct_ values increased in the order of (-)-epigallocatechin < (-)-epicatechin < EGCG, (-)-epicatechin gallate [10]. Therefore, a correlation between partition coefficient and inhibitory effect on P-glycoprotein was not observed. The *n*-octanol/PBS partition coefficient of quercetin, a tea flavonoid, was larger than that of EGCG, but the inhibitory effect on P-glycoprotein of quercetin was smaller than that of EGCG [10]. Therefore, EGCG is relatively hydrophilic, however its amphiphilic chemical structure may be favorable for the interaction with either the substrate binding sites or the activity-modulating sites of P-glycoprotein.

### 3.2. Turmeric

Curcumin is a major component of the culinary spice turmeric (*Curcuma longa*) and it gives specific flavor and a yellow color to curry. Curcumin has strong anti-oxidant and anti-inflammatory properties and is reported to prevent the initiation, promotion, or progression of cancer. Curcumin is one of the most extensively investigated chemopreventive phytochemicals [29]. It is also reported that curcumin is pharmacologically safe. Human clinical trials indicated no dose-limiting toxicity when administered at doses of up to 10 g/day [29].

Curcumin is reported to suppress NF-κB activation induced by TNF, phorbor esters, and hydrogen peroxide through suppression of IκB degradation [6,7,29]. In 1995, Singh and Aggarwal [30] firstly reported that curcumin is a potent inhibitor of NF-κB signaling. They used human myelomonoblastic leukemia cell line ML-1a and electrophoretic mobility shift assay (EMSA) to show the activation of NF-κB in ML-1a cells by TNF, TPA and hydrogen peroxide [30]. Curcumin blocked the TNF-induced phosphorylation and degradation of IκB, and translocation of NF-κB to the nucleus [30]. The activation of NF-κB by TNF, TPA and hydrogen peroxide were inhibited by curcumin [30]. Chun *et al.* [31] also reported that curcumin inhibits the TPA-induced expression of cyclooxygenase-2 in mouse skin through the suppression of NF-κB activation.

The Wnt signaling pathway is also an important target of the cancer preventive activity of curcumin. Jaiswal *et al*. [19] showed that β-catenin-induced c-Myc expression is important in curcumin-induced growth arrest and apoptosis in human colon cancer HCT-116 cells. Park *et al*. [20] have reported the inhibitory mechanism of curcumin using β-catenin/TCF reporter construct. They suggested that the inhibitory mechanism of curcumin is related to the reduced amount of nuclear β-catenin and TCF-4 proteins [20].

The mammalian target of rapamycin (mTOR), a serine/threonine kinase, is downstream in the phosphatidylinositol 3-kinase (PI3K)/Akt (protein kinase B) cascade. mTOR integrates signals regarding nutrient and energy availability, mitogen activation, and thus regulates the cell growth and survival [32]. Recent findings show that curcumin can inhibit mTOR signaling in cancer cells [33,34]. Yu *et al*. [34] have reported curcumin inhibits mTOR signaling in human prostate cancer PC-3 cells through a protein phosphatase-dependent dephosphorylation mechanism.

We have examined the inhibitory effect of curcumin on human P-glycoprotein using P-glycoprotein-overexpressing human carcinoma KB-C2 cells and fluorescent P-glycoprotein substrates [11]. Curcumin increased the accumulation of daunorubicin in KB-C2 cells in a concentration-dependent manner. The cellular accumulation of rhodamine 123 was also increased in the presence of curcumin. Since curcumin inhibits the efflux of P-glycoprotein substrates, the elevation of substrate accumulation seems to be induced by the inhibition of the efflux transporter. The efflux of rhodamine 123 from KB-C2 cells was decreased by curcumin [11]. Taken together, these findings indicate that curcumin has an inhibitory effect on P-glycoprotein function, and may have chemosensitizing potency, in addition to its own chemopreventive properties.

### 3.3. Chili pepper and ginger

Capsaicin is a pungent component of hot and red chili pepper (*Capsicum annuum*). In addition to alleviating pain and itching in humans, capsaicin has exhibited chemopreventive effects, suppressing carcinogenesis of the skin, colon, lung, tongue, and prostate [6,7]. Singh *et al*. [35] have reported the inhibition of TNF-induced NF-κB activation in human myeloid ML-1a cells by capsaicin. Capsaicin blocked the degradation of IκB, and nuclear translocation of NF-κB [35]. Han *et al*. [36] also showed that capsaicin inhibits the TPA-induced activation of NF-κB in mouse skin.

[6]-Gingerol is a phenolic substance responsible for the spicy taste of ginger (*Zingiber officinale*). [6]-Gingerol has also been linked with prevention of cancer. It has been reported that [6]-gingerol inhibits the proliferation of cancer cells including prostate, gastric, and breast [6,7]. Kim *et al.* [37] reported that [6]-gingerol suppresses TPA-induced degradation of IκB and translocation of NF-κB to the nucleus, consequently inhibits the activation of NF-κB. Lee *et al*. [38] recently reported that [6]-gingerol downregulates β-catenin-depenent cyclin D1 levels and induce apoptosis in human colon cancer cells.

Capsaicin and [6]-gingerol have also shown to have inhibitory effects on human P-glycoprotein [11]. Capsaicin and [6]-gingerol increased the intracellular concentration of P-glycoprotein substrates by inhibiting this anticancer drug efflux transporter [11]. In the presence of 50 μM capsaicin or [6]-gingerol, multidrug-resistant carcinoma KB-C2 cells were more susceptible to the cytotoxicity of vinblastine, a P-glycoprotein substrate, as compared with vinblastine alone [11]. This demonstrates that capsaicin and [6]-gingerol can partially reverse multidrug resistance in cells that express P-glycoprotein.

### 3.4. Rosemary

The leaves of rosemary (*Rosmarinus officinalis*) are commonly used as a spice in cooking. However, because of the presence of phenolic diterpenes and triterpenes with strong antioxidative activity, interest has grown in using rosemary as a natural antioxidant in foods [39,40,41,42,43,44,45,46]. Extract of rosemary leaves have been used to prevent lipid autooxidation and to inhibit the oxidation of edible oils or fats. The antioxidative activity of rosemary extract is comparable with that of known antioxidants, such as butylated hydroxytoluene (BHT) and butylated hydroxyanisole (BHA), without the cytotoxic and carcinogenic risk. Among the constituents of rosemary extracts, 90% of the total antioxidative activity was derived from carnosic acid and carnosol [39,40,41,42,43,44]. Rosemary also yields substantial quantities of the polyphenolic antioxidant rosmarinic acid. Rosemary extract is regarded as safe, and a relatively high concentration, 0.02 to 0.05% (w/w), is used for food production [45,46]. A typical commercial rosemary extract contains 20% (w/w) carnosic acid. Therefore, 100 g of meat could contain 30 μmol of carnosic acid [46].

In addition to antioxidative activities, rosemary phytochemicals are reported to have antimicrobial, anti-inflammatory, and anticancer properties [47,48,49,50,51]. Topical application of carnosol and ursolic acid to mouse skin inhibited TPA-induced inflammation and tumorigenesis [47]. Carnosol restricts the invasion of B16/F10 mouse melanoma cells by reducing metalloproteinase-9 activity through inhibition of NF-κB [50]. Carnosol is shown to block the lipopolysaccharide (LPS)-induced phosphorylation and degradation of IκB, and nuclear translocation of NF-κB [48]. Ursolic acid inhibits NF-κB activation induced by various carcinogens [49]. Carnosol is also reported to prevent β-catenin/APC-associated intestinal carcinogenesis [51]. Therefore, rosemary phytochemicals, carnosic acid, carnosol, rosmarinic acid, and ursolic acid, are regarded as strong natural antioxidants and promising dietary chemopreventive agents.

We have examined the effects of rosemary phytochemicals on the function of human P-glycoprotein [52]. Carnosic acid, carnosol, and ursolic acid increased the cellular accumulation of daunorubicin or rhodamine 123 in multidrug-resistant KB-C2 cells. On the other hand, rosmarinic acid had no effect on the accumulation of daunorubicin [52]. We have previously investigated the effects of flavonoids on P-glycoprotein function [53]. The inhibitory effects of flavonoids were in the order of kaempferol > quercetin, baicalein > myricetin > fisetin, morin. Quercetin-3-glycoside and rutin had no effects [53]. These results indicate that the hydrophobicity of phytochemicals is important for their inhibitory effects on the efflux of substrates by P-glycoprotein. Rosmarinic acid, a hydrophilic antioxidant, had no effect on P-glycoprotein [52]. In contrast, ursolic acid, a steroid-like triterpene that has little or no antioxidative activity, inhibited P-glycoprotein function [52]. Therefore, the hydrophobicity, not the antioxidative activity, of phytochemicals could be important for their inhibitory effects on P-glycoprotein activity. We have also examined the effects of rosemary phytochemical on the resistance to vinblastine cytotoxicity. In the presence of as little as 10 μM concentration of carnosic acid, KB-C2 cells were more susceptible to the cytotoxicity of vinblastine, a P-glycoprotein substrate, as compared with vinblastine alone [52]. This demonstrates that rosemary phytochemical carnosic acid has a chemosensitizing effect, reversing P-glycoprotein-mediated multidrug resistance by increasing the intracellular accumulation of anticancer drug.

### 3.5. Citrus fruits

The ingestion of citrus fruit has been reported to be beneficial for the reduction of certain types of human cancer. Citrus phytochemicals, including monoterpenes, limonoids, flavonoids, and coumarins are demonstrated to have anti-carcinogenic effects [54]. Auraptene (7-geranyloxycoumarin), a coumarin-related compound occurring widely in citrus fruit (e.g., grapefruit), markedly inhibited 4-nitroquinoline 1-oxide-induced oral carcinogenesis in rats and suppressed the growth of human prostate carcinoma cells [55,56,57]. Auraptene also showed suppression of TPA- and LPS- induced inflammatory responses [58]. Nobiletin (5,6,7,8,3',4'-hexamethoxyflavone), a polymethoxyflavonoid in citrus fruit (e.g., mandarins and oranges), is an inhibitor of the generation of both NO and O_2_^-^ in leukocytes and showed strong inhibitory effects on TPA-induced skin inflammation, oxidative stress, and tumor promotion in mice and the growth of human prostate carcinoma cells [57,59]. Therefore, citrus phytochemicals, auraptene and nobiletin, are considered promising chemopreventive agents.

The concentration of auraptene is 6.03 μM in grapefruit juice, and the concentration of nobiletin is 6.71 to 11.43 μM in Valencia orange juice [55,60,61,62]. It is reported that these concentrations could be sufficient to inhibit the function of P-glycoprotein [63]. However, further studies of the absorption, distribution, metabolism, and excretion of citrus phytochemicals in the human body are needed to clarify the inhibitory effects of these compounds at the site of drug action, in the tumor tissues and cancer cells.

It is reported that ATP hydrolysis and substrate transport are tightly coupled, and most compounds that are known to be transported by the ABC transporter stimulate ATPase activity [2]. To explore the inhibitory mechanism of citrus phytochemicals on the function of P-glycoprotein, effects on ATPase activity of P-glycoprotein was measured using human P-glycoprotein membranes from baculovirus infected insect cells. The ATPase activity of P-glycoprotein was stimulated by verapamil, a known substrate of P-glycoprotein [63]. Auraptene or nobiletin alone also stimulated the basal ATPase activity of P-glycoprotein [63]. Auraptene and nobiletin further enhanced the verapamil-stimulated ATPase activity of P-glycoprotein [63]. These results suggest that auraptene and nobiletin could be substrates of P-glycoprotein, and competitively interact at drug-binding site of P-glycoprotein.

### 3.6. Plant sterols

Cholesterol is an essential structural component of animal cell membranes. Plant sterols play analogues roles in plants. The major dietary phytosterols are β-sitosterol, campesterol and stigmasterol, and their contents are higher in edible oils, seeds and nuts. Dietary plant sterols and stanols have been recognized as efficient modulators of plasma low-density lipoprotein (LDL) cholesterols in humans for decades [64,65]. The mechanism underlying this hypocholesterolemic effect is a reduction in cholesterol absorption from the intestinal lumen into the circulation. Elevated levels of serum LDL cholesterol is a major cause of cardiovascular disease, and several studies showed that phytosterols reduce coronary heart disease risk [64,65]. Epidemiologic and experimental studies also suggest the role of dietary plant sterols in the protection from cancers, such as colon, breast and prostate cancers [66].

Resin of the gum of the guggul tree (*Commiphora mukul*) has been used to treat hyperlipidemia, obesity, arthritis, and inflammation in humans [67,68]. The active substance from the resin has been demonstrated to be the plant sterol guggulsterone [67,68]. Guggulipid is an extract of the resin, which is enriched for guggulsterone. Several clinical trials demonstrate that guggulipid effectively lowers serum LDL cholesterols levels, although these data have been recently questioned [67,68,69]. Guggulipid is commonly used in India for treatment of hyperlipidemia and obesity and is widely available worldwide as an herbal dietary supplement. The pharmacological activity of guggulsterone has been suggested to be mediated by the antagonism of the nuclear receptor, farnesoid X receptor (FXR, NR1H4) [70,71]. Guggulsterone also suppresses TNF-induced phosphorylation, degradation of IκB and translocation of NF-κB to the nucleus [72]. Consequently, guggulsterone inhibits the activation of NF-κB and expression of anti-apoptotic gene products, and thus enhance apoptosis in human lung carcinoma cells [72].

We have investigated the effects of plant sterols on P-glycoprotein function [73]. β-Sitosterol, campesterol, stigmasterol, β-sitostanol, β-cholestanol and fucosterol had no effects [73]. These results suggest that commonly consumed dietary plant sterols, such as β-sitosterol, campesterol and stigmasterol, do not interact with human P-glycoprotein. In contrast, guggulsterone increased the cellular accumulation of daunorubicin in P-glycoprotein-overexpressing KB-C2 cells in a concentration-dependent manner [73]. Since this phytosterol does not show any effect in P-glycoprotein-lacking KB-3-1 cells, the elevation of substrate accumulation in KB-C2 cells seems to be induced by the inhibition of the anticancer drug efflux transporter. The cellular accumulation of rhodamine 123 was increased, and the efflux of rhodamine 123 from KB-C2 cells was decreased by guggulsterone [73]. Guggulsterone also stimulated the ATPase activity of P-glycoprotein. Therefore, guggulsterone could be a substrate of P-glycoprotein, and competitively inhibit P-glycoprotein at its drug-binding site.

### 3.7. Others

In addition to the above-mentioned phytochemicals, various natural compounds are reported to possess cancer preventive properties [5,6,7]. Several phytochemicals were also studied for their effect on P-glycoprotein [11,74,75]. Resveratrol, a phytoalexin present in grapes (*Vitis vinifera*), sesamin, a lignan existing exclusively and abundantly in sesame (*Sesamum indicum*) seeds, matairesinol found in soybean (*Glycine max*), glycyrrhetinic acid and glabridin in licorice (*Glycyrrhiza glabra*) extract, and ginsenosides and their hydrolyzed metabolites from the root of *Panax ginseng* have been shown for their inhibitory effects on the human P-glycoprotein [11,74,75].

## 4. MRP1 and Chemopreventive Phytochemicals

Multidrug resistance protein 1 (MRP1, ABCC1), encoded by the *ABCC1* gene, belongs to the ABC transporter family, and is the second member to be identified. MRP1 consists of 17 transmembrane domains and two ATP-binding sites, and acts as a drug efflux transporter [3,4]. In contrast to P-glycoprotein, MRP1 expression is widespread in the body, including lung, testis, skeletal and cardiac muscles [3,4]. P-glycoprotein and MRP1 also differ in substrate specificity. Although the mechanisms by which substrates are recognized by P-glycoprotein and MRP1 have not been fully clarified, P-glycoprotein seems to prefer amphipathic cationic compounds, and MRP1, anionic compounds [3,4]. Both P-glycoprotein and MRP1 act as anticancer drug efflux transporters and cause multidrug resistance. It is also reported that P-glycoprotein and MRP1 were major determinants of innate drug sensitivity, even when the level of expression was low in drug-naïve tumors [76]. Inhibitors of P-glycoprotein and MRP1 are useful not only to reverse or prevent acquired drug resistance, but also to sensitize drug-naïve untreated tumors to anticancer drugs. Therefore, MRP1 is also a promising target for the reversal of multidrug resistance and a better outcome of cancer chemotherapy.

Several natural compounds were investigated for their inhibitory effects on MRP1 [52,63,73,74,77,78,79,80,81]. Quercetin, EGCG and curcumin are reported to interact with human MRP1 [77,78,79,80,81]. We have investigated the effects of chemopreventive phytochemicals on the function of MRP1 using human MRP1 gene-transfected KB/MRP cells [52,63,73,74]. None of the rosemary phytochemicals, carnosic acid, carnosol, rosmarinic acid, and ursolic acid, affects the MRP1 activity [52]. Citrus phytochemical nobiletin, but not auraptene, inhibits the function of MRP1 [63]. Dietary plant sterols β-sitosterol, campesterol, stigmasterol, β-sitostanol, β-cholestanol and fucosterol had no effects on MRP1 [73]. Guggulsterone is an inhibitor of both P-glycoprotein and MRP1 [73].

Glycyrrhetinic acid is also a dual inhibitor of P-glycoprotein and MRP1 [74]. It is interesting that the inhibitory mechanisms of glycyrrhetinic acid might be different in the two ABC transporters. Glycyrrhetinic acid alone stimulated the basal ATPase activity of MRP1 [74]. In the presence of NEM-GS, an MRP1 substrate, the ATP hydrolysis by MRP1 was further stimulated by glycyrrhetinic acid. In contrast to the result of MRP1 ATPase activity, glycyrrhetinic acid alone had no effect on the ATPase activity of P-glycoprotein. However, glycyrrhetinic acid inhibited the verapamil-stimulated P-glycoprotein ATPase activity [74]. These results suggest that glycyrrhetinic acid could be a substrate of MRP1, and competitively interact at drug-binding site of MRP1. In contrast, glycyrrhetinic acid is not a substrate of P-glycoprotein, but possibly interacts noncompetitively at ATP hydrolytic site of P-glycoprotein. In the presence of 100 μM concentration of glycyrrhetinic acid, KB-C2 and KB/MRP cells were more susceptible to the cytotoxicity of vinblastine, a P-glycoprotein substrate, or doxorubicin, an MRP1 substrate, as compared with vinblastine or doxorubicin alone [74]. This demonstrates that glycyrrhetinic acid has a chemosensitizing effect, reversing P-glycoprotein and MRP1-mediated multidrug resistance by increasing the intracellular accumulation of anticancer drug.

## 5. Future Perspective

Although P-glycoprotein and MRP1 are key determinants of drug sensitivity, other anticancer drug efflux transporters, such as breast cancer resistance protein (BCRP, ABCG2), can cause multidrug resistance [3,4]. At present, interactions between natural compounds and anticancer drug efflux transporters, other than P-glycoprotein and MRP1, have not been well investigated. Therefore, it is important to study the inhibitory effects of phytochemicals on BCRP and other ABC transporters.

In the studies using cancer cell lines, relatively higher doses of phytochemicals are often used. Even though the natural compounds are regarded as safe, a low amount is favorable for future *in vivo* studies. It is noteworthy to consider that the level of P-glycoprotein expression in the cell line is much greater than that in human tissues [2,3,4]. Thus, lower concentrations of phytochemicals would be effective in modulating *in vivo* P-glycoprotein activities. In addition, synergistic effects with other dietary phytochemicals on ABC transporter function should be considered. Combinatorial therapy with several natural compounds and conventional anticancer drugs must be developed for the future cancer chemotherapy.

As described in this review, cellular signaling pathways, such as NF-κB and Wnt, are important molecular targets of cancer prevention by natural compounds. Recent studies have revealed that P-glycoprotein is also regulated by these pathways [82]. β-Catenin/TCF binding sites in the *MDR1* promoter were described previously and recently it has been shown that the activation of Wnt/β-catenin signaling increases P-glycoprotein expression [83,84,85,86]. In contrast, suppression of Wnt/β-catenin signaling decreased P-glycoprotein expression [85,86]. Natural compounds that inhibit both of P-glycoprotein function and expression can be useful for overcoming multidrug resistance in human cancer. Therefore, I am now trying to find such compounds by using both P-glycoprotein-lacking anticancer drug sensitive cells and P-glycoprotein-overexpressing multidrug-resistant cells.

It is also very important to investigate the absorption, distribution, metabolism, and excretion of chemopreventive phytochemicals in the human body, to clarify the interaction with anticancer drugs at the site of drug action, in the tumor tissues and cancer cells.

## 6. Conclusion

In addition to the beneficial effects of natural compounds, the inhibitory effects of phytochemicals on the anticancer drug efflux transporter P-glycoprotein and MRP1 have been revealed, which can reverse the multidrug resistance. Therefore, dietary chemopreventive phytochemicals, such as nobiletin, guggulsterone and glycyrrhetinic acid, can be considered promising lead compounds for the design of more efficacious and less toxic chemosensitizing agents to enhance the efficacy of chemotherapy in cancer patients.
